# Sensing of heavy metal Pb^2+^ ions in water utilizing the photonic structure of highly controlled hexagonal TiON/TiO_2_ nanotubes

**DOI:** 10.1038/s41598-023-50428-2

**Published:** 2024-01-10

**Authors:** Asmaa M. Elsayed, Ashour M. Ahmed, M. T. Tammam, M. F. Eissa, Arafa H. Aly

**Affiliations:** 1 Nanophotonics and Applications Lab, Physics Department, Faculty of Science, Beni-Suef University, Beni-Suef, 62514 Egypt; 2 TH-PPM Group, Physics Department, Faculty of Science, Beni-Suef University, Beni Suef, 62514 Egypt; 3 Physics Department, Faculty of Science, Beni-Suef University, Beni Suef, 62514 Egypt; 4 Physics Department, College of Science, Imam Mohammad Ibn Saud Islamic University (IMSIU), 11623 Riyadh, Saudi Arabia

**Keywords:** Materials science, Optics and photonics, Physics

## Abstract

The detection of heavy metals in water, especially Pb^2+^ ions, is important due to their severe hazardous effects. To address this issue, a highly controlled hexagonal TiON/TiO_2_ heterostructure has been synthesized in this study. The fabrication process involved the utilization of atomic layer deposition and direct current sputtering techniques to deposit TiO_2_ and TiON layers onto a porous Al_2_O_3_ membrane used as a template. The resulting heterostructure exhibits a well-ordered hollow tube structure with a diameter of 345 nm and a length of 1.2 µm. The electrochemical sensing of Pb^2+^ ions in water is carried out using a cyclic voltammetry technique under both light and dark conditions. The concentration range for the Pb^2+^ ions ranges from 10^–5^ to 10^–1^ M. The sensitivity values obtained for the sensor are 1.0 × 10^–6^ in dark conditions and 1.0 × 10^–4^ in light conditions. The remarkable enhancement in sensitivity under light illumination can be attributed to the increased activity and electron transfer facilitated by the presence of light. The sensor demonstrates excellent reproducibility, highlighting its reliability and consistency. These findings suggest that the proposed sensor holds great promise for the detection of Pb^2+^ ions in water, thereby facilitating environmental monitoring, water quality assessment, and safety regulation across various industries. Furthermore, the eco-friendly and straightforward preparation techniques employed in its fabrication provide a significant advantage for practical and scalable implementation.

## Introduction

Heavy metals are a significant concern for both human health and the environment. These elements can easily enter the human body through various pathways, including internal sources such as contaminated water and food, as well as external sources in the ambient environment^1–3^. This ease of transfer makes heavy metal contamination a serious concern for public health and environmental safety^4,5^. When heavy metals leach into water bodies or soil, they can contaminate the food chain. For instance, plants may absorb these metals from the soil, and then animals consume the contaminated plants, leading to bioaccumulation in their tissues. Eventually, humans may be exposed to these toxic metals by consuming contaminated plants or animals^6–8^. In addition to direct ingestion, heavy metals can also be inhaled through the air, particularly in industrial areas where emissions from factories and vehicles release metal particles into the atmosphere. This can lead to inhalation of airborne particulates containing heavy metals, further increasing the risk of exposure.

The impact of heavy metal exposure on human health varies depending on the specific metal and the level of exposure. Heavy metals such as lead, mercury, cadmium, and arsenic are known to be highly toxic and can result in a wide range of health issues. These may include neurological disorders, organ damage, developmental abnormalities, and even cancer. Therefore, it is crucial to prioritize the development of sensitive and selective sensors for the detection and quantification of these metals in the environment and various matrices (such as water, soil, and food)^9,10^. Lead is a highly toxic substance that can affect humans. It is released into the environment through various human activities, such as burning fossil fuels, mining operations, and manufacturing processes. Lead is extensively used in industries including the production of lead-acid batteries, ammunition, metal products, X-ray shielding devices, paint oxides, glass, pigments, sheet lead, ceramic products, and pipe solder. In the USA, approximately 1.52 million metric tons of lead were used for industrial applications in 2004. Unfortunately, lead poisoning has become a significant concern, particularly for children. As a result, children living in homes with lead exposure can have blood lead concentrations of 20 µg/dL or highe^11^. Lead toxicity affects various organs in the body, including the kidneys, liver, central nervous system, hematopoietic system, endocrine system, and reproductive system. Among these, the central nervous system is particularly vulnerable to the effects of lead poisoning.

Today, sensors are becoming a vital component in environmental monitoring systems, providing valuable insights into heavy metal contamination and contributing to sustainable resource management and pollution control efforts. The detection and quantification of heavy metals present significant challenges for scientists and researchers. Various analytical techniques, such as gas chromatography, mass spectrometry, dispersive X-ray fluorescence spectrometry, and laser ablation have been employed for this purpose. However, these techniques often involve high costs and complex preparation processes^12,13^. As a result, scientists are aiming to explore alternative sensing techniques that provide a balance of affordability, environmental friendliness, and ease of preparation. One promising approach is the development of porous thin film membrane sensors based on highly active materials. These sensors are designed to be simple, low-cost, and easy to prepare. The use of thin film technology allows for efficient sensing of heavy metals, as it provides a large surface area for interaction with the target ions. This high surface area enhances the sensitivity and responsiveness of the sensor, making it suitable for detecting trace amounts of heavy metals^14–16^. Another advantage of thin film membrane sensors is their portability and versatility. These sensors can be integrated into various devices, such as handheld detectors or wearable sensors, enabling on-site and real-time monitoring of heavy metal levels. Their ease of use and rapid response make them valuable tools for field applications, where timely detection is critical for preventing potential hazards^17,18^.

The porous Al_2_O_3_ membrane proves to be a highly promising approach for the preparation of nanomaterials with well-defined shapes and sizes^19,20^. The porous Al_2_O_3_ membrane was fabricated by electrochemical oxidation (anodization) of high-purity aluminum in acidic electrolytes^21^. The precise control over pore size, shape, spacing, and thickness during the anodization process allows for tailored properties and enhanced functionalities. This membrane exhibits excellent chemical properties, including high chemical stability and resistance to corrosion. It also possesses good mechanical strength and thermal stability, enabling its use in harsh environments and integration into diverse devices and systems. Moreover, this Al_2_O_3_ membrane is chemically inert and non-toxic, making it suitable for biomedical applications. Also, it possesses optical transparency in the visible and near-infrared range, enabling effective manipulation and control of light^22^. This characteristic makes this template well-suited for the development of optical devices like waveguides, filters, and lenses. The ordered pore arrangement in this template can act as a photonic crystal, enabling the engineering of photonic bandgaps and manipulation of light propagation. By depositing thin films or nanoparticles onto the surface of this membrane template, additional functionalities such as plasmonic effects or enhanced light scattering can be introduced. Moreover, by preserving the open pores and depositing nanomaterial on the top surface of this template, the increased surface area creates more sites for chemical reactions and adsorption^23^. One of the key advantages of using the Al_2_O_3_ membrane is the generation of nanomaterials with highly active sites, leading to increased reactivity. The combination of precise morphology control, high activity sites, and porous thin film behavior makes the Al_2_O_3_ membrane a valuable tool in the design and synthesis of advanced nanomaterials with tailored properties. These properties open up new applications for nanomaterials with high performance in various industries. For example, the preparation of TiO_2_ thin films using the Al_2_O_3_ membrane opens up opportunities in fields such as sensors, electronic devices, and coatings.

Our team possesses supplementary literature focusing on the identification of hazardous heavy metals, particularly Hg^2+^, Cd^2+^, and Pb^2+^. This literature explores the use of highly sensitive nanomaterials, specifically WO_2_I_2_/polypyrrole, poly(m-toluidine), and poly(m-cresol). The sensitivity for Pb^2+^ ions was initially measured at 0.4 µA/M with the poly(m-toluidine) material^24^ and subsequently elevated to 1.1 µA/M when employing the poly(m-cresol)/Pt sensor^25^.

Herein, the fabrication process involves using the Ni-imprinting technique to obtain highly controlled hexagonal-shaped Al_2_O_3_ membranes. These membranes serve as containers for the deposition of TiO_2_ and TiN nanomaterials. This structure (TiON/TiO_2_/Al_2_O_3_) is then utilized as a sensor for the detection of Pb^2+^ ions in aqueous solutions. The TiON/TiO_2_ combination provides enhanced reactivity and catalytic capabilities, facilitating the electrochemical sensing of Pb^2+^ ions. The Al_2_O_3_ membrane acts as a stable and robust support for the nanomaterials, ensuring their longevity and performance during the sensing process. The sensing techniques are studied under a wide range of concentrations from 10^–4^ to 10^–1^ M using the cyclic voltammetry (CV) technique.

## Experimental methods

### Materials

Al foil (99.9%) was obtained from Sigma-Aldrich, USA. Ethylene glycol (C_2_H_6_O_2_) and chromic acid (H_2_CrO_4_) were acquired from Sigma Aldrich, USA. Perchloric acid (HClO_4_), ethanol (C2H5OH), phosphoric acid (H_3_PO_4_), and titanium tetrachloride (TiCl_4_) were obtained from the VWR company in Germany. N2 and Ar gases are obtained from El-Naser Company, Egypt.

### Characterization of synthesized materials

Various techniques were employed to analyze the structural composition properties of the Al_2_O_3_ membrane and TiON/TiO_2_. Energy dispersive X-ray spectroscopy (EDAX; Oxford Link ISIS 300) was utilized to accurately determine the chemical elements present in the samples. X-ray diffraction (XRD) analysis was conducted using a Panalytical (Empyrean) X-ray diffractometer with CuKα radiation (λ = 0.154 nm), applying 40 kV and 35 mA, and measuring 2θ values within the range of 25°–80°. Morphological analysis was performed using a transmission electron microscope (TEM; JOEL JEM-2010) as well as a scanning electron microscope (SEM; Axioskop 40 POL by Zeiss).

### ***Synthesizing of TiON/TiO***_***2***_*** hexagonal using Al***_***2***_***O***_***3***_*** membrane***

The synthesis of the Al_2_O_3_ membrane involves the Ni imprinting technique. The utilization of the Ni-imprinting technique enables the transfer of a hexagonal pattern onto the Al foil, facilitating the creation of an Al_2_O_3_ membrane with a range of desired shapes, such as square, triangular, diamond, and hybrid pattern pores. This technique involves applying pressure using a Ni mold before anodization, leading to the successful replication of the chosen shape on the surface of the Al foil.

A hexagonal Ni mold is used to imprint its shape onto Al foil (purity 99.99%) under a pressure of 10 kN/cm^2^. The Al foil is then subjected to electropolishing in a two-electrode cell using an electrolyte solution of HClO_4_ and C_2_H_5_OH (1:1) at a temperature of 2 °C for 3 min. Subsequently, the first and second anodization processes are carried out at the same temperature. The anodization was performed for 10 min followed by an additional 30 min using an electrolyte solution of H_3_PO_4_, ethylene glycol, and H_2_O in a ratio of 1:100:200. Afterward, the etching process was conducted using a solution of H_3_PO_4_ (6 wt.%) and H_2_CrO_4_ (1.2 wt.%) at a temperature of 60 °C for 12 h. Finally, the pore widening process is performed using H_3_PO_4_ (6 wt.%) at 60 °C for 30 min to achieve the desired pore size,

TiO_2_ deposition is achieved through the atomic layer deposition (ALD) technique, in which this technique offers several advantages such as high-quality films, low-temperature processing, precise control over film thickness and stoichiometry, scalability for large-scale production, excellent repeatability, and low defect. For the preparation of TiO_2_, the utilization of TiCl_4_ and H_2_O as precursor sources at 300 °C. The deposition process was carried out for 300 cycles to ensure an adequate TiO_2_ thickness. On the other hand, TiON is prepared using DC magnetron sputtering. A mixture of N_2_ and Ar gases with concentrations of 75 and 25 sccm, respectively, was used during the deposition process. A Ti target is utilized, and the deposition is performed under a low pressure of 13 × 10^–3^ mbar. The actual deposition time of TiN is about few minutes, but the total time for this procedures takes about four h. Finally, the TiON film was subjected to combustion at 300 °C for 2 h to complete the film preparation.

### The potentiometric sensing

The TiON/TiO_2_/Al_2_O_3_ hexagonal heterostructure is utilized as a potentiometric sensor for the detection of Pb^2+^ ions. The sensing process is conducted using a three-electrode cell, where the TiON/TiO_2_ heterostructure serves as the working electrode. The intensity of the cyclic voltammetry signal corresponds to the concentration of Pb^2+^ ions and provides information about the sensor's sensitivity towards Pb^2+^ ions. This testing is carried out under both dark and light conditions to examine the influence of photon illumination on the sensing performance using the CHI608E workstation. The TiON/TiO_2_ heterostructure serves as the main electrode. The Pt sheet and Ag/AgCl serve as counter and reference electrodes.

### Ethics approval

This article does not contain any studies involving animals or human participants performed by any of the authors.

## Results and discussion

### Analyses

**Figure 1:**
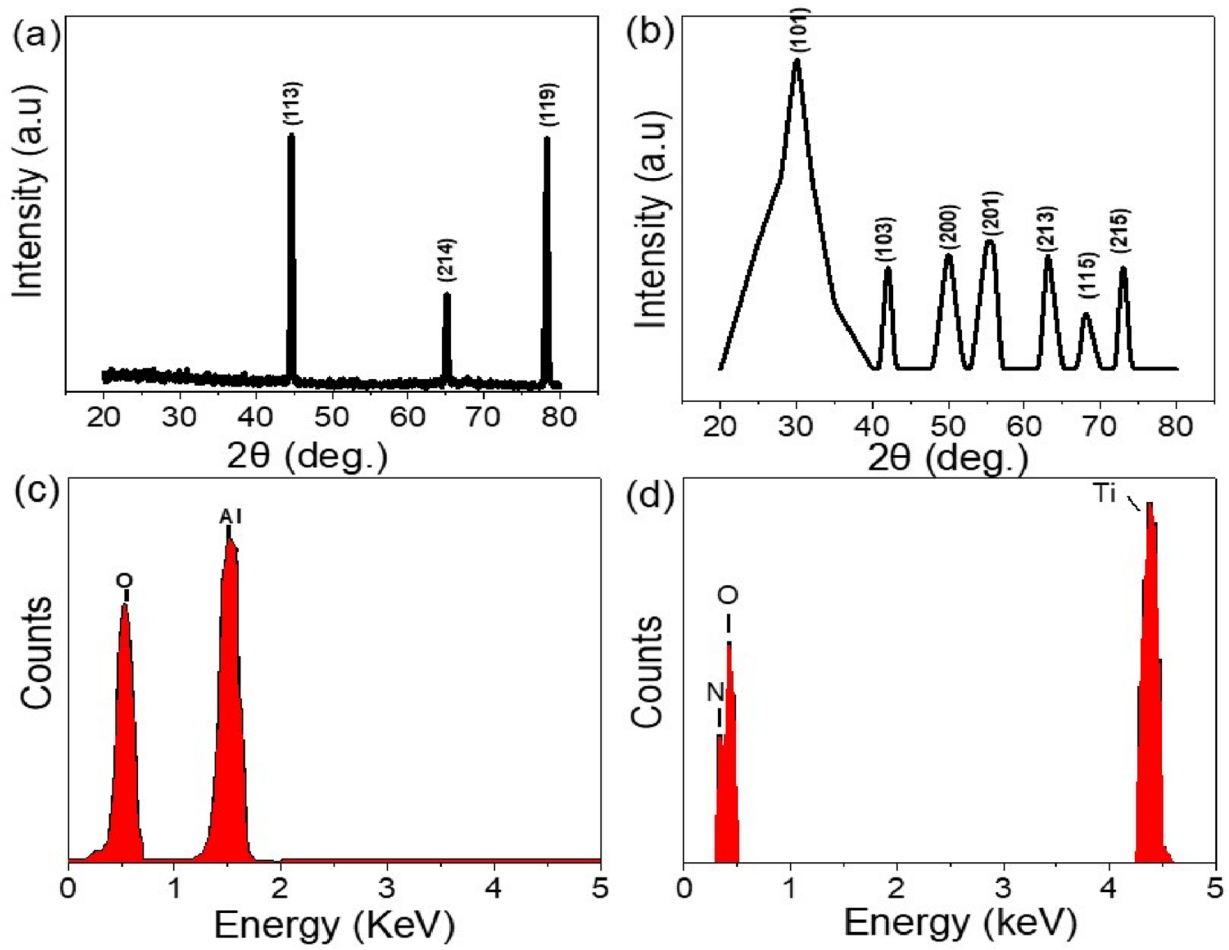
The XRD analyses of (**a**) Al2O3 membrane and (**b**) TiON/TiO_2_ heterostructure. EDX analyses of (**c**) Al_2_O_3_ membrane and (**d**) TiON/TiO2 heterostructure.

The XRD pattern of Al_2_O_3_ in Fig. 1a confirms the crystalline nature of the Al_2_O_3_ nanomaterial. This is evident from the appearance of three sharp peaks located at 47.7°, 65.1°, and 78.3°, corresponding to the growth directions (113), (214), and (119), respectively^26^. On the other hand, the XRD pattern of the TiON/TiO_2_ heterostructure shows the presence of seven sharp peaks as seen in Fig. 1b. These peaks are located at 30.0°, 42.1°, 50.0°, 55.4°, 63.1°, 68.1°, and 73.2° correspond to the Miller indices (101), (103), (200), (201), (213), (115), and (215), respectively. These peaks are characteristic of TiO_2_ nanomaterials. The TiON does not appear to have distinct peaks in the XRD chart due to the TiON layer in the heterostructure is very thin (approximately 5 nm) and may not exhibit a well-defined crystalline structure.

The EDX analyses of prepared nanomaterials provide comprehensive insights into the material's elemental composition, as depicted in Fig. 1. The EDX analyses conducted on Al_2_O_3_, as depicted in Fig. 1c, provide conclusive evidence of the presence of oxygen (O) and aluminum (Al) elements in the material. This indicates that the prepared Al_2_O_3_ membrane exhibits high purity without the presence of any significant impurities. This finding agrees with the XRD analysis, further supporting the assertion that the Al_2_O_3_ material is of high quality and exhibits the expected chemical composition with good crystallinity. Similarly, for the TiON/TiO_2_ heterostructure, as demonstrated in Fig. 1d, the presence of titanium (Ti), oxygen (O), and nitrogen (N) elements is confirmed. The EDX spectrum shows characteristic peaks corresponding to these elements, indicating their presence in the heterostructure material.

**Figure 2:**
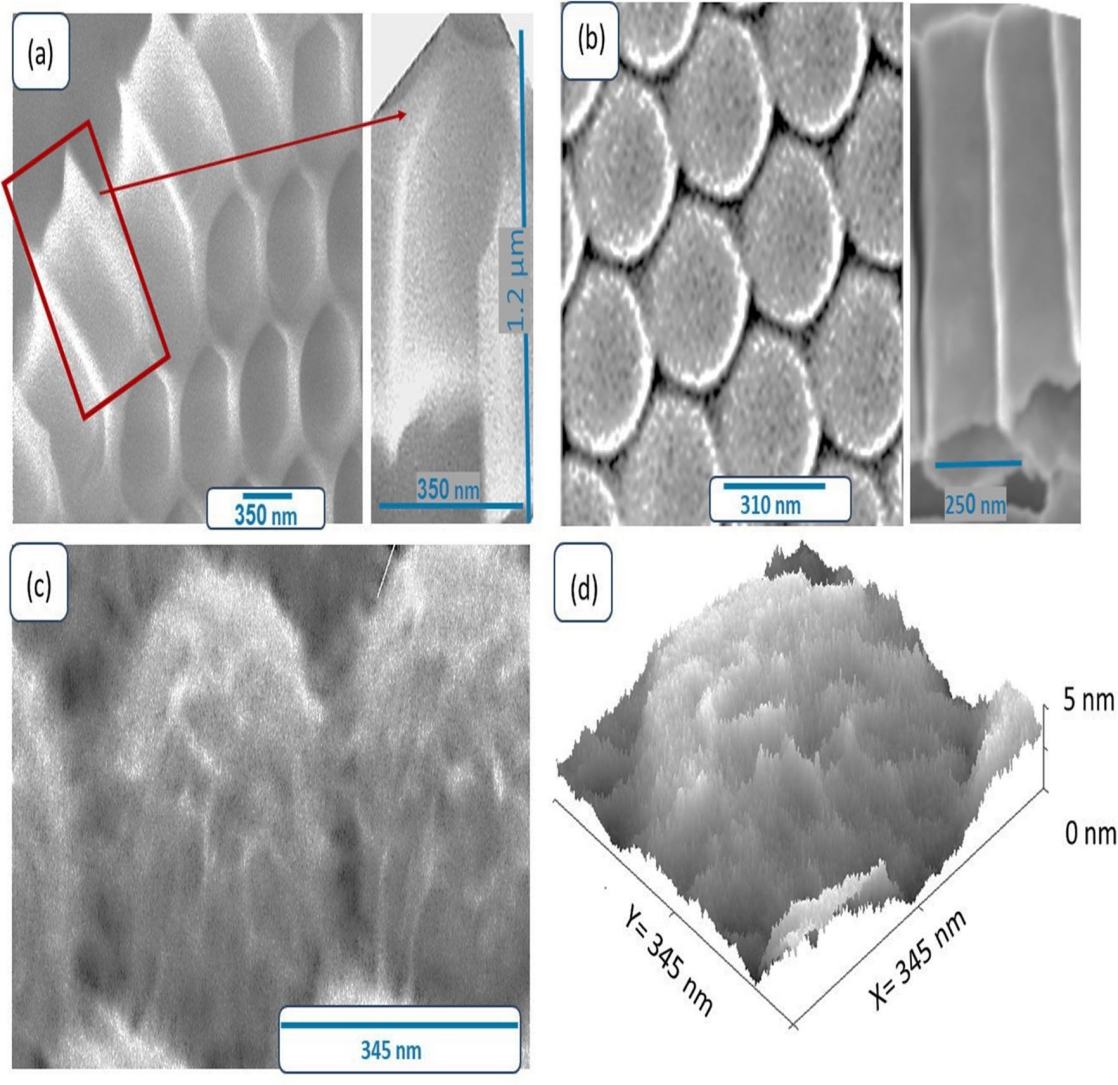
SEM image of (**a**) Al_2_O_3_ membrane (top-view and cross section), (**b**) TiO2 face and cross-section, (**c**) TiON/TiO_2_, and (**d**) Cross section roughness of TiON/TiO2.

The Al_2_O_3_ membrane displayed in Fig. 2a exhibits remarkable morphological and topographical properties. It features a distinct porous hexagonal shape like a bee house with a diameter of 350 nm. The large diameter of the porous ensures a significant amount of analyte molecules can be accommodated within the nanotubes, facilitating enhanced sensing performance. Upon closer examination of the magnified image of Fig. 2a, the depth of the Al_2_O_3_ membrane is approximately 1.2 µm. This depth indicates that the membrane possesses adequate thickness to support subsequent material depositions, ensuring stability and mechanical integrity. Additionally, the substantial length of 1.2 µm provides ample room for functionalization or modification with specific sensing elements or surface functional groups, further tailoring their sensing capabilities. This characteristic is crucial for the successful synthesis of functional nanomaterials and nanostructures based on the membrane. The regularity and uniformity of the hexagonal pattern provide an ideal scaffold for further material deposition or growth, enabling the creation of functional nanostructures with tailored properties. This high-order membrane was fabricated using the Ni-imprinting technique, which has shown promise in synthesizing membranes with precise and well-defined structures^27,28^. The Ni-imprinting technique involves the use of a nickel mold pressed onto the Al_2_O_3_ surface under controlled conditions. This process enables the replication of intricate patterns with high fidelity and precision. After the nickel mold is removed, the desired hexagonal bee-house structure remains embedded in the Al_2_O_3_, ready for further exploration and applications.

Figure 2b presents the TiO_2_ layer deposited on the Al_2_O_3_ membrane. It is characterized by high-order tubes with a diameter of 310 nm and a length of 1.2 µm. The TiO_2_ nanotubes have unique properties that make them highly attractive for various technological and scientific pursuits including environmental monitoring, healthcare diagnostics, and industrial quality control. The hollow structure possesses several advantages for sensing applications due to the abundance of active sites present both inside and outside their walls. The highly active sites present within the walls of the TiO_2_ nanotubes create an environment conducive to sensing interactions, resulting in rapid and efficient detection of target analytes. Also, the hollow structure allows for efficient diffusion of analytes and enhances the surface area available for interactions. The combination of active sites and high surface area allows for efficient binding and recognition of analyte molecules, promoting improved sensitivity and response.

The deposition of TiON on the TiO2/Al_2_O_3_ results in the formation of a highly fibrous structure that covers the TiO_2_ nanotubes, as shown in Fig. 2c. This fibrous morphology, with a width of approximately 5 nm, contributes to the increased roughness of the heterostructure material. The roughness of the TiON/TiO_2_ heterostructure is illustrated in Fig. 2d using the Gwydion program. The presence of irregularities and rough textures amplifies the available surface area and the number of active sites for interactions with analyte molecules. Additionally, the presence of roughness fibers on the surface of the TiON/TiO_2_ heterostructure can have implications for light absorption due to multiple reflections and trapping light. Under light conditions, the increased roughness and active sites provide even greater opportunities for photon-induced charge separation. This can be beneficial for sensing applications, as it facilitates stronger sensing responses and improved sensitivity to target analytes, particularly under light conditions.

### Sensing properties

The sensing performance of the TiON/TiO_2_ heterostructure sensor towards Pb^2+^ ions is evaluated using the cyclic voltammetry technique. The Pb^2+^ ions are tested across a concentration range of 10^–5^ to 10^–1^ M in a three-electrode cell configuration. The investigations were conducted under both dark and light conditions. A metal halide lamp serves as the light source for illumination with a power intensity of about 100 mW/cm^2^. The photon illumination plays a crucial role in initiating surface interactions within TiON/TiO_2_ semiconductor materials.

The incorporation of the TiON layer into the TiON/TiO2 heterostructure offers numerous advantages for sensor performance. Firstly, it acts as a protective layer, safeguarding the underlying materials from environmental degradation and ensuring long-term stability. Additionally, the TiON layer introduces roughness textures, resulting in a larger surface area and an increased number of active sites. This, in turn, provides more opportunities for absorption and interactions with analyte molecules. The presence of roughness fibers on the TiON layer facilitates efficient light interaction by enabling multiple reflections and light trapping, thereby maximizing the chances for photon-induced charge separation. Ultimately, these benefits significantly enhance the sensitivity of the sensor in detecting target analytes or signals, particularly under light conditions.

**Figure 3:**
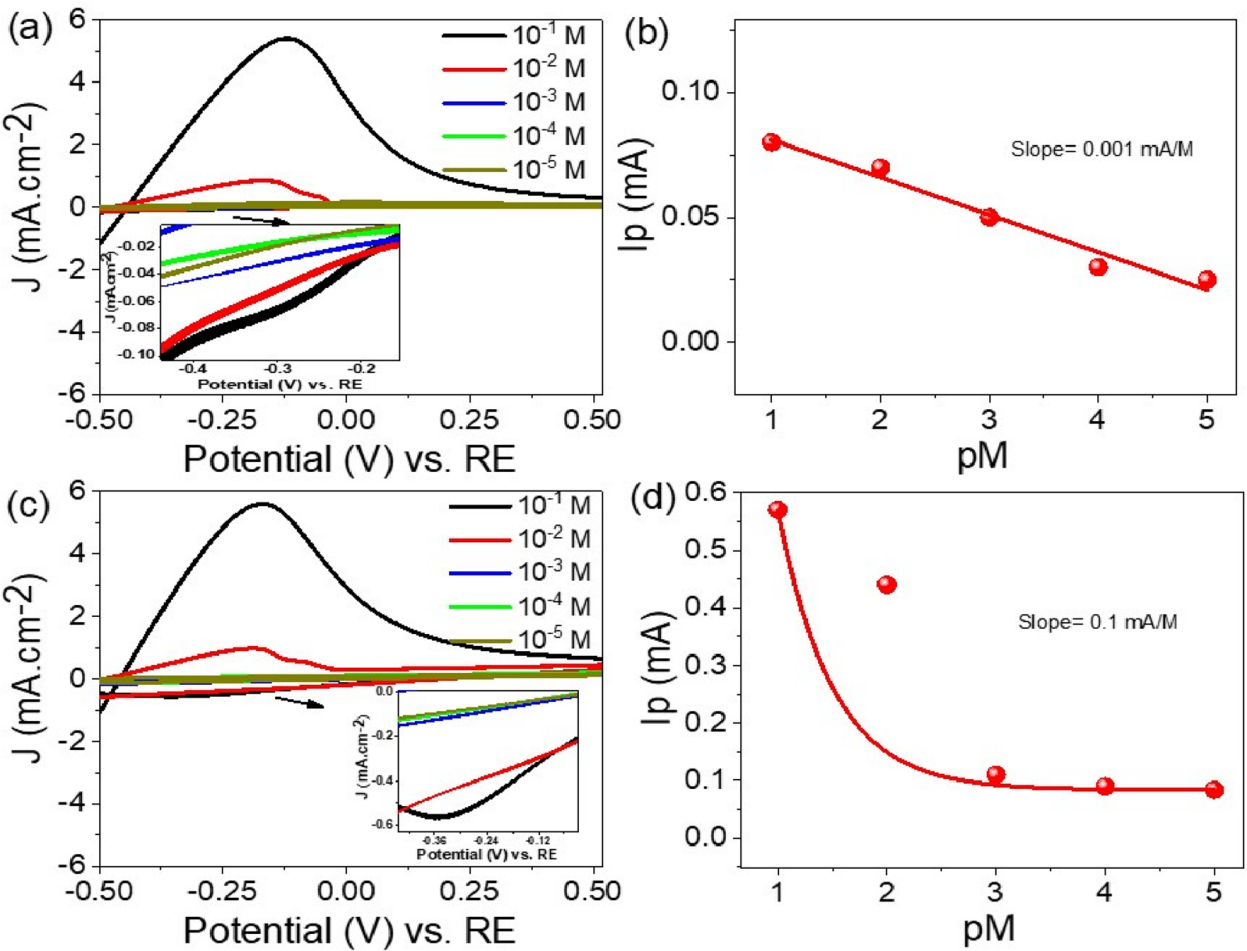
(**a**, **c**) The cyclic voltammetry technique for Pb^2+^ ion electrolyte with varying concentrations from 10–5 to 10–1 M (**b**, **d**) and Corresponding peak current of the cyclic voltammogram against the pM value (negative logarithm of the Pb^2+^ ion concentration) in dark condition under light.

Figure 3a presents the cyclic voltammogram (CV) with the varying concentration of Pb^2+^ ions. The main sensing mechanism relies on electrostatic attraction between the sensor and Pb^2+^ ions present in the solution. In this case, the CV is performed under dark conditions, which means there is no light influence on the electrochemical reactions. The main reduction peaks for CV are illustrated at − 0.28 V. The area of CV increases with the rising concentration of Pb^2+^ ions from 10^–5^ to 10^–1^ M. The main observation is that the area under the CV curve increases with increasing concentration of Pb^2+^ ions. The area under the curve is related to the amount of charge exchanged during the electrochemical reaction. As the concentration of Pb^2+^ ions increases, the reduction peaks in the CV also tend to increase in magnitude. This is because as the Pb^2+^ ion concentration increases, more Pb^2+^ ions are available to participate in the reduction reaction at the electrode surface during the CV experiment. Consequently, a larger reducing current response is generated, resulting in higher peaks in the CV. This behavior indicates the ability of the TiON/TiO_2_ heterostructure to detect and respond to varying levels of Pb^2+^ ions in the tested solution^29^. To evaluate the sensitivity of the sensor, the pM value (negative logarithm of Pb^2+^ ion concentration) is plotted against the corresponding peak current, as shown in Fig. 3b. The rate of change in the peak current with respect to the concentration of Pb^2+^ ions is determined by linear fitting for experimental data. It is about 1 × 10^–6^ A/M based on this analysis. This value represents the sensitivity of the TiON/TiO_2_ heterostructure for the electroanalytical detection trace of Pb^2+^ ions in aqueous solutions. The obtained result demonstrates the potential of the TiON/TiO_2_ heterostructure as an efficient and reliable sensor in environmental monitoring and analytical applications.

Figure 3c shows data of the CV for the detection of Pb^2+^ ions under light illumination. The TiON/TiO_2_ heterostructure sensor exhibits a significant enhancement in the reduction peaks compared to the dark condition when exposed to light. This increase in reduction peaks indicates the positive impact of light on improving the sensing behavior. The light intensity facilitates a faster and more efficient response to the presence of Pb^2+^ ions in the solution. Under light conditions, the TiON/TiO_2_ semiconductor materials are activated by photon energy, leading to the excitation of electrons to higher energy levels. Consequently, an excess of electrons accumulates on the surface of the TiO_2_/TiON photoelectrode. Hence, the TiO_2_/TiON photoelectrode acts as an efficient electron donor^30–32^. Moreover, the incorporation of the TiON layer brings about surface roughness, leading to an expanded surface area with an augmented quantity of active sites. Consequently, this creates a greater number of possibilities for the absorption and interaction of analyte molecules. The existence of rough fibers on the TiON layer promotes effective light interaction by allowing for multiple reflections and light trapping, ultimately maximizing the opportunities for photon-induced charge separation. Overall, these advantages substantially elevate the sensitivity of the sensor when it comes to detecting Pb^2+^ analytes. The accumulation of electrons creates a favorable environment for electrostatic interactions with Pb^2+^ ions, resulting in their adsorption onto the sensor material through strong electrostatic forces. The light-induced photocatalytic effect significantly amplifies the sensing performance of the sensor, enabling it to detect trace levels of Pb^2+^ ions in the solution. In contrast, under dark conditions, the electron excitation process is not activated, and the accumulation of charge carriers on the surface is limited. As a result, the sensor sensitivity is low to detect Pb^2+^ ions. The relationship between the peak current and the concentration of Pb^2+^ ions follows an exponential behavior rather than a linear one, as depicted in Fig. 3d. This characteristic further emphasizes the significant influence of light on the performance of the TiON/TiO_2_ heterostructure sensor. The exponential relationship indicates that the sensing process is highly sensitive to light illumination, offering the advantage of fine-tuning the sensor performance under light^33^. The sensor demonstrates a sensitivity of approximately 1 × 10^–4^ A/M, which is higher than that observed under dark conditions. By leveraging the power of light activation, this sensor holds great promise as a precise and efficient tool for detecting Pb^2+^ ions, with potential applications in environmental monitoring.

**Figure 4:**
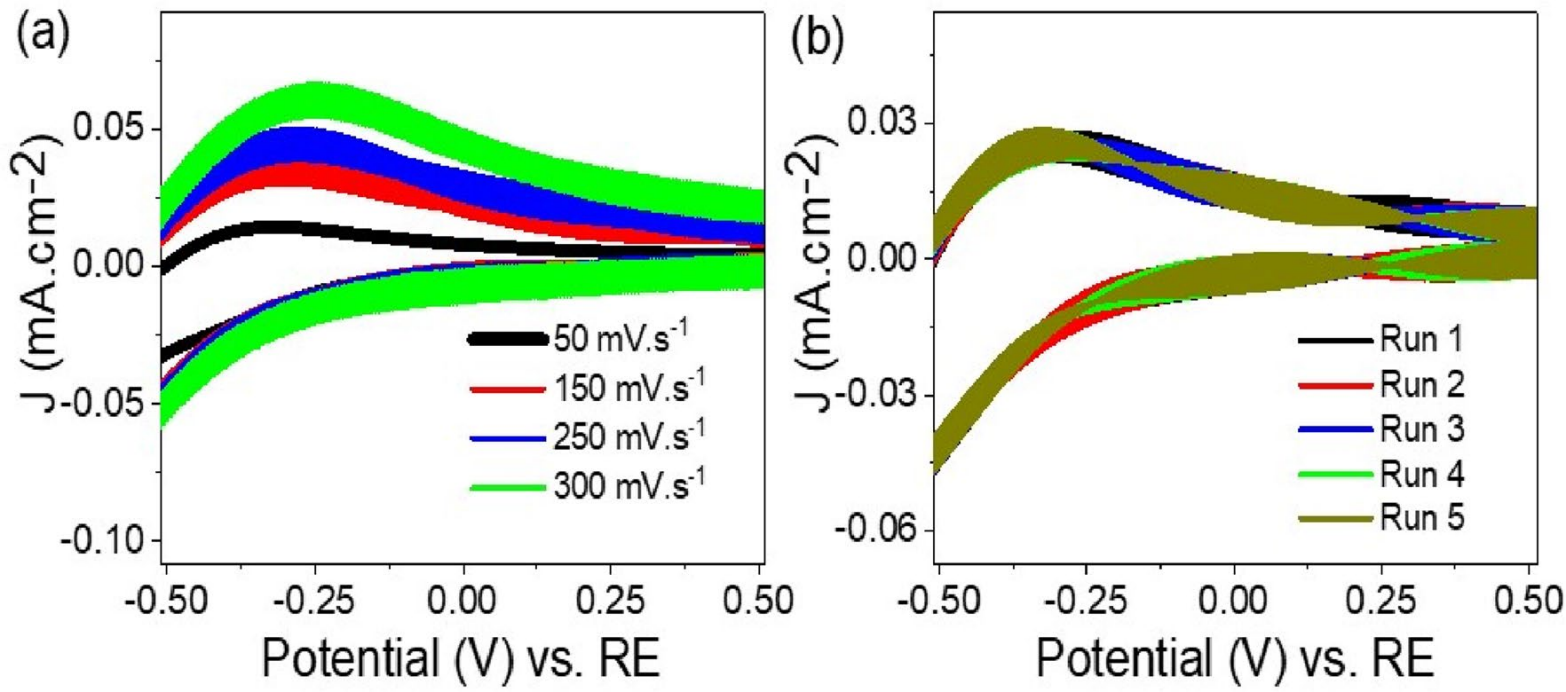
The effect of (**a**) scan rate from 50 to 300 mV s-1and (**b**) reproducibility (five runs) of the Pb^2+^ ion detection by TiON/TiO_2_ heterostructure sensor that estimated through the cyclic voltammetry technique under dark conditions.

The effect of scan rate on the sensing process is crucial in determining its efficiency. The scan rate represents the speed at which the potential is changed during CV measurements. The sensitivity of the TiON/TiO_2_ heterostructure sensor towards Pb^2+^ ions at a concentration of 10^–3^ M was studied by varying the scan rate. Figure 4a displays the results of the study, where the scan rate was varied in the range of 50 to 300 mV/s. It was observed that the cyclic curve showed enhancement with higher scan rates. This indicates that as the scan rate is increased, the movement of Pb^2+^ ions towards the TiON/TiO_2_ heterostructure sensor also increases. Consequently, there is a corresponding increase in the peak current observed in the cyclic voltammogram^25^. The increase in peak current with higher scan rates demonstrates the sensitivity of the TiON/TiO_2_ heterostructure sensor in detecting Pb^2+^ ions at low concentrations.

To evaluate the stability of the TiON/TiO_2_ heterostructure sensor's response to Pb^2+^ ions, the CV measurements were repeated several times at a concentration of 10^–3^ M. Figure 4b displays the results obtained from five consecutive runs under light. The results demonstrate that the TiON/TiO_2_ heterostructure sensor maintains a consistent current value with negligible variation. This remarkable stability indicates that the sensor's sensing behavior remains unchanged over multiple cycles, without any discernible performance degradation or drift. In other words, the sensor exhibits high sensitivity and reliability throughout the repetitive measurements, ensuring reliable and accurate detection of Pb^2+^ ions. The stability demonstrated by the TiON/TiO_2_ sensor is crucial for practical applications, especially in long-term monitoring and real-time detection scenarios^2,34,35^. The ability to maintain consistent and reproducible results over multiple measurement cycles makes the sensor a reliable and robust tool for electroanalytical detection of Pb^2+^ ions. The results highlight that the TiON/TiO_2_ heterostructure sensor possesses a favorable combination of eco-friendliness, affordability, high sensitivity, and reproducibility, making it a suitable solution for detecting Pb^2+^ ions in drinking water within industrial settings. This advancement in water management significantly contributes to the improved protection of public health.

**Figure 5:**
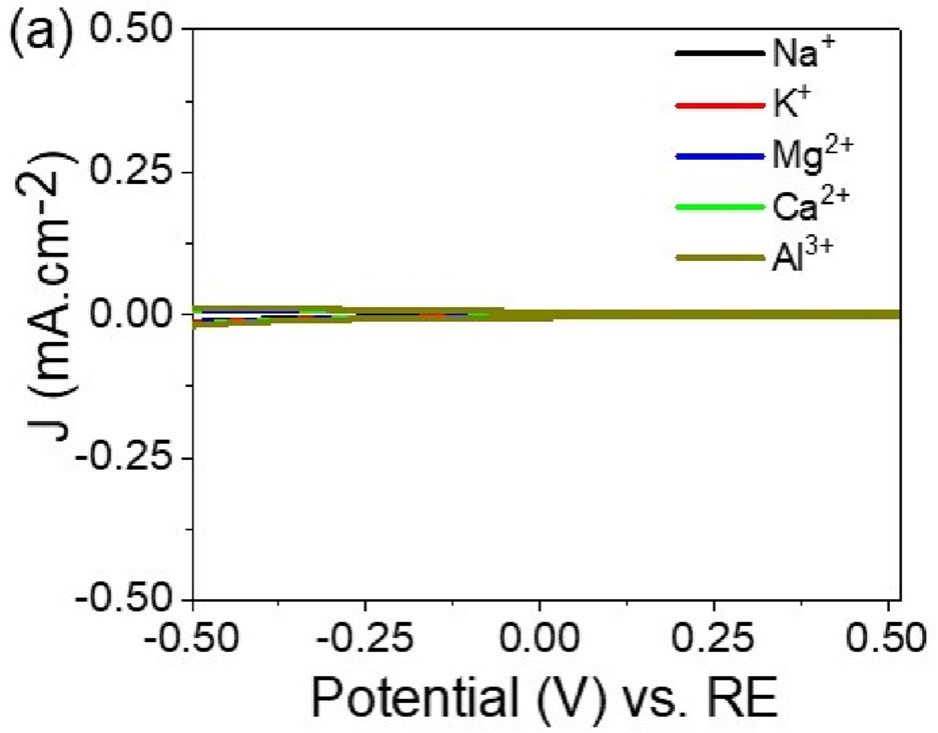
The cyclic voltammetry of TiON/TiO2 heterostructure sensor for some interfering ions (Na^+^ , K^+^ , Mg2^+^ , Ca2^+^ , and Al^3+^).

Figure 5 illustrates the impact of interfering ions, including Na^+^, K^+^, Mg^2+^, Ca^2+^, and Al^3+^ at a concentration of 0.01 M, on the sensitivity of the developed TiON/TiO_2_ heterostructure sensor. The absence of discernible oxidation or reduction peaks for these ions in the figure suggests that there is negligible interference from Na^+^, K^+^, Mg^2+^, Ca^2+^, and Al^3+^ on the sensitivity of the TiON/TiO_2_ heterostructure sensor. This lack of observable peaks indicates that these ions do not impede the sensor's ability to detect Pb^2+^ ions effectively. The graph in Fig. 5 serves as evidence that the fabricated TiON/TiO2 heterostructure sensor remains selective in its response to Pb^2+^ ions in the presence of the specified interfering ions. This outcome is crucial for the reliability and accuracy of the sensor in real-world applications where diverse ionic environments may be encountered. Consequently, the TiON/TiO_2_ heterostructure sensor exhibits promising potential for precise and selective detection of Pb^2+^ ions in the presence of common interfering ions, making it a valuable tool for environmental monitoring and analytical applications.

## Conclusions

Using the atomic layer deposition (ALD) technique, a hexagonal nanotube TiON/TiO_2_ heterostructure has been successfully created on a porous Al_2_O_3_ membrane. This heterostructure displays a well-organized hollow tube design with a diameter of 345 nm and a length of 1.2 µm. The application of TiON on the TiO_2_/Al_2_O_3_ substrate leads to a highly fibrous structure, increasing active sites and surface area, and facilitating effective light interaction. Consequently, this heterostructure shows promise for sensing applications. Specifically, the TiON/TiO_2_ membrane exhibits outstanding electrochemical sensing capabilities for Pb^2+^ ions due to its high surface charge density. The electrostatic attraction between the sensor and Pb^2+^ ions results in heightened sensitivity, particularly in the presence of light. For electrochemical sensing of Pb^2+^ ions in water, a cyclic voltammetry device is utilized under both light and dark conditions, with Pb^2+^ ion concentrations ranging from 10^–5^ to 10^–1^ M. Sensitivity values obtained from cyclic voltammetry for the sensor are 1.0 × 10^–6^ in dark conditions and 1.0 × 10^–4^ in light conditions. The notable increase in sensitivity under light is attributed to enhanced activity and efficient electron transfer facilitated by light. The proposed sensor's practical and scalable nature positions it as an appealing solution for widespread use in environmental monitoring, water quality assessment, and safety regulation. Its accurate and timely detection capabilities for heavy metal contamination in water systems empower the implementation of effective monitoring and mitigation strategies.

## Data Availability

Requests for materials should be addressed to Arafa H. Aly.

## References

[1] Hamid Kargari S, Ahour F, Mahmoudian M (2023). An electrochemical sensor for the detection of arsenic using nanocomposite-modified electrode. Sci. Rep..

[2] Ebrahim S, Shokry A, Khalil MMA, Ibrahim H, Soliman M (2020). Polyaniline/Ag nanoparticles/graphene oxide nanocomposite fluorescent sensor for recognition of chromium (VI) ions. Sci. Rep..

[3] Hossain NI, Tabassum S (2023). A hybrid multifunctional physicochemical sensor suite for continuous monitoring of crop health. Sci. Rep..

[4] Lan T, Fallatah A, Suiter E, Padalkar S (1944). Size controlled copper (I) oxide nanoparticles influence sensitivity of glucose biosensor. Sensors.

[5] Zhan W, Chen Z, Hu J, Chen X (2018). Vertical CuO nanowires array electrodes: Visible light sensitive photoelectrochemical biosensor of ethanol detection. Mater. Sci. Semicond. Process..

[6] Hadia NMA, Abdelazeez AAA, Alzaid M, Shaban M, Mohamed SH, Hoex B, Hajjiah A, Rabia M (2022). Converting sewage water into H2 fuel gas using Cu/CuO nanoporous photocatalytic electrodes. Materials.

[7] El-Rahman AMA, Rabia M, Mohamed SH (2023). Nitrogen doped TiO2 films for hydrogen generation and optoelectronic applications. J. Mater. Sci.: Mater. Electron..

[8] Abdelazeez AAA, Hadia NMA, Mourad AHI, El-Fatah GA, Shaban M, Ahmed AM, Alzaid M, Cherupurakal N, Rabia M (2022). Effect of Au plasmonic material on poly M-toluidine for photoelectrochemical hydrogen generation from sewage water. Polymers.

[9] Leoci R, Ruberti M, Leoci R, Ruberti M (2021). Pesticides: An overview of the current health problems of their use. J. Geosci. Environ. Protection.

[10] Azizi-Toupkanloo H, Karimi-Nazarabad M, Shakeri M, Eftekhari M (2019). Photocatalytic mineralization of hard-degradable morphine by visible light-driven Ag@g-C3N4 nanostructures. Environ. Sci. Pollut. Res..

[11] Tchounwou PB, Yedjou CG, Patlolla AK, Sutton DJ (2012). Heavy metals toxicity and the environment. EXS.

[12] Cai LM, Wang QS, Wen HH, Luo J, Wang S (2019). Heavy metals in agricultural soils from a typical township in Guangdong Province, China: Occurrences and spatial distribution. Ecotoxicol. Environ. Saf..

[13] Gopal J, Hasan N, Manikandan M, Wu HF (2013). Bacterial toxicity/compatibility of platinum nanospheres, Nanocuboids Nanoflowers. Sci. Rep..

[14] Sabah FA, Ahmed NM, Hassan Z, Almessiere MA (2017). Influence of CuS membrane annealing time on the sensitivity of EGFET PH sensor. Mater. Sci. Semicond. Process..

[15] Khalil MM, Issa YM, Zayed SIM, Ali FQ (2014). New potentiometric membrane sensors for determination of alverine citrate in pharmaceutical compounds and biological fluids. Int. J. Adv. Res..

[16] Altowyan AS, Shaban M, Gamel A, Gamal A, Ali M, Rabia M (2022). High-performance PH sensor electrodes based on a hexagonal Pt nanoparticle array-coated nanoporous alumina membrane. Materials.

[17] Sayyah SM, Shaban M, Rabia M (2016). A high-sensitivity potentiometric mercuric ion sensor based on m-toluidine films. IEEE Sens. J..

[18] Sayyah SM, Shaban M, Rabia M (2016). A sensor of M-toluidine/m-cresol polymer film for detection of lead ions by potentiometric methods. Sensor Lett..

[19] Mi Y, Zhou M, Wen L, Zhao H, Lei Y (2014). A highly efficient visible-light driven photocatalyst: Two dimensional square-like bismuth oxyiodine nanosheets. Dalton Transact..

[20] Mohamed SH, Zhao H, Romanus H, El-Hossary FM, Abo EL-Kassem M, Awad MA, Rabia M, Lei Y (2020). Optical water splitting and wettability of titanium nitride/titanium oxynitride bilayer films for hydrogen generation and solar cells applications. Mater. Sci. Semicond. Process..

[21] Ahmed AM, Shaban M (2020). Nanoporous chromium thin film for active detection of toxic heavy metals traces using surface-enhanced raman spectroscopy. Mater. Res. Express.

[22] Shaban M, Ahmed AM, Abdel-Rahman E, Hamdy H (2016). Morphological and optical properties of ultra-thin nanostructured Cu films deposited by RF sputtering on nanoporous anodic alumina substrate. Micro Nano Lett..

[23] Eessaa AK, El-Shamy AM (2023). Review on fabrication, characterization, and applications of porous anodic aluminum oxide films with tunable pore sizes for emerging technologies. Microelectron. Eng..

[24] Sayyah SM, Shaban M, Rabia M (2016). A sensor of M-toluidine/m-cresol polymer film for detection of lead ions by potentiometric methods. Sensor Letters.

[25] Sayyah ESM, Shaban M, Rabia M (2018). A sensor of M-cresol nanopolymer/Pt-electrode film for detection of lead ions by potentiometric methods. Adv. Polymer Technol..

[26] Al-Taweel SS, Saud HR (2016). New route for synthesis of pure anatase TiO 2 nanoparticles via utrasound-assisted sol-gel method. J. Chem. Pharm. Res..

[27] Zhan Z, Grote F, Wang Z, Xu R, Lei Y, Zhan Z, Grote F, Wang Z, Xu R, Lei Y (2015). Degenerating plasmonic modes to enhance the performance of surface plasmon resonance for application in solar energy conversion. Adv. Energy Mater..

[28] Wang Z, Cao D, Wen L, Xu R, Obergfell M, Mi Y, Zhan Z, Nasori N, Demsar J, Lei Y (2016). Manipulation of charge transfer and transport in plasmonic-ferroelectric hybrids for photoelectrochemical applications. Nat. Commun..

[29] El-Fatah GA, Magar HS, Hassan RYA, Mahmoud R, Farghali AA, Hassouna MEM (2022). A novel gallium oxide nanoparticles-based sensor for the simultaneous electrochemical detection of Pb2+, Cd2+ and Hg2+ ions in real water samples. Sci. Rep..

[30] Zhong F, Wang H, Wang Z, Wang Y, He T, Wu P, Peng M, Wang H, Xu T, Wang F (2021). Recent progress and challenges on two-dimensional material photodetectors from the perspective of advanced characterization technologies. Nano Res..

[31] Sreedhar A, Ta QTH, Noh J-S (2023). Versatile role of 2D Ti3C2 MXenes for advancements in the photodetector performance: A review. J. Ind. Eng. Chem..

[32] Tan M, Hu C, Lan Y, Khan J, Deng H, Yang X, Wang P, Yu X, Lai J, Song H (2017). 2D lead dihalides for high-performance ultraviolet photodetectors and their detection mechanism investigation. Small.

[33] Shaban M, Ali S, Rabia M (2019). Design and application of nanoporous graphene oxide film for CO2, H2, and C2H2 gases sensing. J. Mater. Res. Technol..

[34] El Nady J, Shokry A, Khalil M, Ebrahim S, Elshaer AM, Anas M (2022). One-Step electrodeposition of a polypyrrole/NiO nanocomposite as a supercapacitor electrode. Sci. Rep..

[35] Khalil MM, Issa YM, El Sayed GA (2015). Modified carbon paste and polymeric membrane electrodes for determination of hydroxychloroquine sulfate in pharmaceutical preparations and human urine. RSC Adv..

